# Elusive sources of variability of dystrophin rescue by exon skipping

**DOI:** 10.1186/s13395-015-0070-6

**Published:** 2015-12-01

**Authors:** Maria Candida Vila, Margaret Benny Klimek, James S. Novak, Sree Rayavarapu, Kitipong Uaesoontrachoon, Jessica F. Boehler, Alyson A. Fiorillo, Marshall W. Hogarth, Aiping Zhang, Conner Shaughnessy, Heather Gordish-Dressman, Umar Burki, Volker Straub, Qi Long Lu, Terence A. Partridge, Kristy J. Brown, Yetrib Hathout, John van den Anker, Eric P. Hoffman, Kanneboyina Nagaraju

**Affiliations:** Research Center for Genetic Medicine, Children’s National Health System, 111 Michigan Avenue N.W., Washington, DC, 20010 USA; Institute of Biomedical Sciences, The George Washington University, Washington, DC, USA; The John Walton Muscular Dystrophy Research Centre, MRC Centre for Neuromuscular Diseases at Newcastle, Institute of Genetic Medicine, Newcastle University, Newcastle upon Tyne, UK; McColl-Lockwood Laboratory for Muscular Dystrophy Research, Neuromuscular/ALS Center, Department of Neurology, Carolinas Medical Center, Charlotte, NC USA; Center for Translational Science, Children’s National Health System, Washington, DC, USA

**Keywords:** Duchenne muscular dystrophy, Dystrophin, Exon skipping, Variability, *mdx-23*

## Abstract

**Background:**

Systemic delivery of anti-sense oligonucleotides to Duchenne muscular dystrophy (DMD) patients to induce de novo dystrophin protein expression in muscle (exon skipping) is a promising therapy. Treatment with Phosphorodiamidate morpholino oligomers (PMO) lead to shorter de novo dystrophin protein in both animal models and DMD boys who otherwise lack dystrophin; however, restoration of dystrophin has been observed to be highly variable. Understanding the factors causing highly variable induction of dystrophin expression in pre-clinical models would likely lead to more effective means of exon skipping in both pre-clinical studies and human clinical trials.

**Methods:**

In the present study, we investigated possible factors that might lead to the variable success of exon skipping using morpholino drugs in the *mdx* mouse model. We tested whether specific muscle groups or fiber types showed better success than others and also correlated residual PMO concentration in muscle with the amount of de novo dystrophin protein 1 month after a single high-dose morpholino injection (800 mg/kg). We compared the results from six muscle groups using three different methods of dystrophin quantification: immunostaining, immunoblotting, and mass spectrometry assays.

**Results:**

The triceps muscle showed the greatest degree of rescue (average 38±28 % by immunostaining). All three dystrophin detection methods were generally concordant for all muscles. We show that dystrophin rescue occurs in a sporadic patchy pattern with high geographic variability across muscle sections. We did not find a correlation between residual morpholino drug in muscle tissue and the degree of dystrophin expression.

**Conclusions:**

While we found some evidence of muscle group enhancement and successful rescue, our data also suggest that other yet-undefined factors may underlie the observed variability in the success of exon skipping. Our study highlights the challenges associated with quantifying dystrophin in clinical trials where a single small muscle biopsy is taken from a DMD patient.

**Electronic supplementary material:**

The online version of this article (doi:10.1186/s13395-015-0070-6) contains supplementary material, which is available to authorized users.

## Background

Duchenne muscular dystrophy (DMD) is one of the most common and severe forms of muscle disease caused by the loss of the dystrophin protein in patients’ muscles [1–9]. Dystrophin-deficient DMD patients show a progressive clinical course, with increasing weakness of the skeletal, cardiac, and respiratory muscles leading to a loss of ambulation in the second decade and early death unless ventilation support is introduced [3, 10, 11]. The most commonly used pharmacological option for DMD patients is daily high-dose corticosteroid treatment [12, 13]. Although daily glucocorticoids prolong ambulation by 2–3 years, they also cause extensive side effect profiles that detract from patients’ quality of life [14, 15].

A therapeutic approach currently in multiple clinical trials in DMD is drug-induced de novo dystrophin expression using exon skipping with anti-sense oligonucleotides (AOs) in the muscle of patients [16–23]. This approach partially repairs the patient’s dystrophin messenger (mRNA) by restoring the triplet codon reading frame, enabling translation of the patient’s RNA [20, 24, 25]. Human clinical trial data for exon skipping in DMD patients remain limited, but the few muscle biopsy data published thus far show highly variable dystrophin expression in patients’ muscle samples. Cirak and colleagues have shown strong immunoblotting and immunostaining evidence of therapeutic levels of dystrophin (>10 %) in only one patient out of 12 following systemic exon 51-skipping AO treatment [20]. Other studies to date have either not reported dystrophin rescue data [26] or the data were challenging to interpret [25, 27].

The exon skipping approach has been extensively studied in pre-clinical models of DMD, including the *mdx* mouse model and the canine X-linked muscular dystrophy (CXMD) model [17, 19, 22, 23, 28, 29]. In the animal model studies, multiple dosing regimens have been tested, and several muscles have been studied in the same treated animal. The results have shown striking variability in the success of the approach between individual myofibers in the same muscle, between different muscle groups in the same animal, and between different animals receiving the same dosing regimen [18, 21]. These pre-clinical findings suggest that there are one or more factors influencing the success of exon skipping, even in adjacent myofibers. Furthermore, the factors driving this variability in pre-clinical models may also be important in human clinical trials, explaining the marked variability in the limited human patient data presented to date.

Pre-clinical data have shown that there is a strong dose effect of morpholino chemistry, with high levels of oligonucleotide drug leading to greater de novo dystrophin production overall [18]. The most successful dystrophin replacement in the *mdx* mouse model has been seen with intravenous bolus doses of 960 mg/kg [30], and CXMD dog studies have shown up to 20 % dystrophin replacement with 200 mg/kg/week delivered intravenously (three AOs simultaneously) [19]. It has been argued that the lack of metabolism of morpholino drugs (they are excreted intact in the urine) and the mechanism of drug delivery via unstable myofiber membranes lead to dose equivalency across species boundaries (e.g., murine dose = human dose) [31]. Most human clinical trials have used doses up to 50 mg/kg/week, suggesting that human trials remain at the low end of the doses needed to see robust de novo dystrophin production. Higher doses have not been attempted in human clinical trials, likely because of both the high cost of the morpholino chemistry [32] and the recommendations of regulatory agencies, with 10-fold higher concentrations being required to be tested thoroughly in rodent models for signs of toxicity.

Understanding the factors that cause the highly variable de novo dystrophin expression seen in pre-clinical models would likely lead to more effective means of exon skipping in both pre-clinical studies and human clinical trials. While the molecular basis for this variability is still unclear, we have recently described that muscle inflammation is linked to the production of TNF-alpha-induced microRNAs that target the dystrophin mRNA and could potentially influence the success of exon skipping in DMD [33].

In the present study, we sought to define other possible factors that might lead to variable success of exon skipping using morpholino drugs in the *mdx* mouse model. Here, a single high bolus of morpholino (800 mg/kg) was administered intravenously (IV) in the *mdx* mouse model. We compared the results for six different muscles using three different methods of dystrophin quantification: immunostaining (or immunofluorescent staining), immunoblotting, and mass spectrometry assays. We then determined whether specific muscle groups or fiber types showed better success than others and finally correlated residual drug concentrations in muscle with the amount of de novo dystrophin protein. Our data suggest that regardless of the quantification method utilized for assessment, the muscle group, fiber type, and residual drug concentration were not well correlated with de novo dystrophin production. These results suggest that other factors may be responsible for the variability observed in the success of exon skipping.

## Methods

### Animals

All animal procedures were conducted in accordance with guidelines for the care and use of laboratory animals as approved by the Institutional Animal Care and Use Committee (IACUC) of Children’s National Health System.

The *mdx* (C57BL/10ScSn-mdx/J) mouse model of DMD, utilized for all experiments, harbors a nonsense point mutation in exon 23 of the dystrophin gene and lacks dystrophin expression in muscle tissue. Four-week-old male *mdx* (*n* = 6) and wild-type (WT) C57BL/10 (*n* = 2) mice were purchased from the Jackson Laboratory (Bar Harbor, ME). All animals were housed at the Children’s National Health System (CNHS) Animal Facility in a vented cage system under 12-h light/dark cycles. Standard mouse chow and water were provided ad libitum.

### Administration of phosphorodiamidate morpholino oligomer

Mice were anesthetized using 4 % isoflurane and 0.5 L/min 100 % oxygen and then maintained using 2 % isoflurane and 0.5 L/min oxygen delivered via a nose cone with a passive exhaust system on a warming device [34]. The phosphorodiamidate morpholino oligomer (PMO) mExon 23(+07-18) (5′- GGCCAAACCTCGGCTTACCTGAAAT- 3′) against the boundary sequences of exon and intron 23 of the mouse dystrophin gene was synthesized by Gene Tools (Philomath, OR, USA). PMO was administered via a single 800 mg/kg dose through an IV injection via the retro-orbital sinus as previously described [35]. PMO was administered in a volume of 300 μl in saline at an injection rate of 2 μl/s (2 min total injection time). After the injection, the mouse was placed back into its cage for recovery and monitored for pain or distress. Control *mdx* mice were injected with 300 μl saline exactly as described for the PMO-treated mice. Uninjected WT C57BL/10 mice were used as dystrophin-positive controls.

### Tissue collection for various quantification methodologies

Mice were sacrificed 1 month after administration of PMOs. Mice were euthanized via carbon dioxide inhalation, and multiple muscle tissues were harvested (tibialis anterior, gastrocnemius, triceps, quadriceps, heart, and diaphragm) [36]. Muscle tissues were quickly removed surgically, cut into three parts, snap-frozen in liquid nitrogen-cooled isopentane, and stored at −80 °C for further analysis. For immunofluorescent staining, muscles were placed on cork, coated with OCT mounting medium, and frozen in liquid nitrogen-cooled isopentane.

### Immunofluorescent staining

#### Dystrophin protein expression

Frozen muscle tissues were sectioned at 10-μm thick and stored at −20 °C until used. Immunofluorescent (IF) for dystrophin protein was performed as described previously [37]. In brief, the muscle sections were brought to room temperature (RT) but not fixed. For dystrophin staining, unfixed sections were blocked with 10 % normal sheep serum, followed by incubation overnight at 4 °C in a humidified chamber with a P7 dystrophin antibody (1:400; Fairway Biotech, England). The P7 antibody binds to the rod domain (exon 57) of the dystrophin protein. Next, the sections were washed and probed with goat anti-rabbit IgG Alexa 594 antibody (1:300; Life Technologies, Grand Island, NY, USA) at RT for 1 h and counterstained with 4′,6-diamidino-2-phenylindole (DAPI) for nuclear staining. The stained tissue sections were stored at 4 °C for further imaging and quantification analyses. Staining was confirmed using alternative dystrophin antibody (Genetex, Irvine, CA, USA). Images were acquired using an Olympus BX61 microscope with attached Olympus DP71 camera module. The surface area of each section and the relative proportion of the dystrophin-positive fiber area were determined using ImageJ software.

### Muscle fiber type

Muscle fiber types were identified using the following antibodies: mouse IgG2b monoclonal anti-type 1 MHC (clone BA-D5, 1:50), mouse IgG1 monoclonal anti-type 2a MHC (clone SC-71, 1:50), mouse IgM monoclonal anti-type 2b MHC (clone BF-F3, 1:5), and mouse IgG1 monoclonal anti-embryonic MHC (clone F1.652, 1:25), all obtained from the Developmental Studies Hybridoma Bank at the University of Iowa (Ames, IA, USA) [38]. Sections were double-stained with dystrophin antibody (Genetex).

In brief, serial cross sections (10-μm thick) were fixed in −20 °C acetone for 10 min. Sections were warmed to RT for 5 min and then incubated in phosphate-buffered saline (PBS) for 15 min, followed by a 1-h incubation in PBS with 0.5 % bovine serum albumin (BSA), 0.5 % Triton X-100, and 1 % horse/goat serum. After three 5-min washes with PBS, samples were incubated for 2 h with primary antibody. After three further 5-min washes with PBS with 0.1 % Tween-20, the samples were incubated for 1.5 h with secondary antibody at 1:500 dilution: Alexa 488-conjugated anti-mouse IgG Fc 2b (for type 1 fibers), Alexa 488-conjugated anti-mouse IgG Fc 1 (for type 2a and embryonic fibers), and Alexa 488-conjugated anti-mouse IgM (for type 2b fibers) (Invitrogen, Carlsbad, CA, USA). Samples were then washed three times for 10 min each, and the slides were mounted using Prolong Gold with DAPI (Life Technologies). Images were acquired using the Olympus BX61 VS virtual slide system (VS120-S5) with attached Olympus XM10 monochrome camera and Olympus VS-ASW FL 2.7 software.

### Immunoblotting (IB) for dystrophin protein expression

Total protein was extracted from the frozen tissues (tibialis anterior, gastrocnemius, triceps, quadriceps, heart, and diaphragm muscles) using radioimmunoprecipitation assay buffer (RIPA) buffer (50 mm Tris-HCl, pH 8.0, with 150 mm sodium chloride, 1.0 % Igepal CA-630 (Nonidet P-40), 0.5 % sodium deoxycholate, and 0.1 % sodium dodecyl sulfate) (Teknova, Hollister, CA, USA) containing protease inhibitors (Halt protease inhibitor mixture 100X; Thermo Fisher Scientific, Waltham, MA, USA). Protein concentrations in the muscle lysates were estimated using the Bio-Rad Microplate Protein Assay (Bio-Rad, Hercules, CA, USA) according to the manufacturer’s protocol.

Extracted proteins from *mdx*-saline (50 μg), *mdx*-PMO (50 μg), and C57BL/10 muscles (3.125 μg) were separated on a Tris-acetate 3–8 % gel (Life Technologies) and transferred overnight at 4 °C onto nitrocellulose membranes. Membranes were blocked using 5 % milk in TBS-Tween (0.1 % Tween) and incubated overnight at 4 °C with DYS1 and DYS2 monoclonal antibodies (1:1000; Leica Microsystems, Buffalo Grove, IL, USA). Membranes were then washed and probed with polyclonal rabbit anti-mouse HRP antibody (1:3000; DAKO, Carpinteria, CA, USA) for 1 h at room temperature. Next, the membranes were incubated with ECL Western Blotting Substrate (GE Healthcare, Piscataway, NJ, USA) and developed on X-ray film (Denville Scientific, South Planfield, NJ, USA). Similarly, membranes were probed with anti-vinculin (1:5000; Abcam Inc, Cambridge, MA, USA) and used as loading controls. Densitometric quantification of band intensity was measured using Quantity One software. The band area to be quantified was determined by identifying the area of the major dystrophin species band, which was kept constant between lanes for an individual blot for analysis. Any possible degradation products were not included in the quantification, as shown in Additional file 1B.

Dystrophin quantifications in morpholino-treated *mdx* muscle were calculated as follows:

percentage of dystrophin expression = (OD from mdx sample/OD from C57BL/10) × dilution factor = 16 (50 μg *mdx*/3.125 μg C57BL/10).

IB runs were performed three times. In the first run, the same amount of total protein was loaded for the WT and *mdx* samples (50 μg). For runs 2 and 3, WT samples were serially diluted to 3.125 μg total protein for loading.

### Mass spectrometry for dystrophin protein expression

Dystrophin protein levels for the tibialis anterior, gastrocnemius, and triceps muscles were determined using MS for PMO-treated *mdx* mice (*n* = 6) in comparison to a C57BL/10 control, as described previously [39]. Using the same protein extracts as for the immunoblots, 50 μg of total protein for each muscle was mixed with 25 μg of an internal standard for stable isotope labeling of amino acid in mammals (SILAM) that had been extracted in the same RIPA buffer from a gastrocnemius muscle [40, 41]. A SILAM mouse is a C57BL/6J mouse fully labeled with ^13^C_6_-lysine, so that all lysine residues are 6 Da heavier [39, 40]. Unlabeled and labeled protein mixtures were separated by 1D electrophoresis. The region corresponding to approximately 300–500 kDa was excised and in gel-digested with trypsin. The resulting peptides were dried by vacuum centrifugation and resuspended in 20 μl of HPLC-grade water with 0.1 % formic acid and 2 % acetonitrile (buffer A). Each sample (5 μl) was injected onto a NanoEasy HPLC and loaded and equilibrated in Buffer A at 800 Bar onto an EasySpray C18 50 μm column, followed by a gradient of 0–35 % acetonitrile at 300 nL/min over 24 min, and coupled online to a Q Exactive mass spectrometer (ThermoFisher, San Jose, CA). The Q Exactive was operated in timed targeted MS2 mode for 13 unlabeled and labeled peptides with the following parameters: positive polarity; resolution 17,500; AGC 1e6; max IT 60 ms; MSX count 4; isolation width 2 m/z; first m/z 150; and NCE 27.

Timed targeted mass spectral data were analyzed using Skyline, version 2.6.0.6709 (skyline.gs.washington.edu) to determine the ratio of unlabeled to labeled for each transition for each peptide. A total of 13 dystrophin peptides and 3 filamin C peptides with four to seven y-ion transitions each were monitored. Peptides with poor co-elution transitions were removed (Skyline “Peptide Peak Found Ratio” score <0.9). Peptide ratios were averaged to give the mean protein ratio. The dystrophin ratio was compared to the filamin C ratio for each sample. PMO-treated samples were compared to the corresponding C57BL/10 muscle to determine the percentage of normal.

### ELISA for PMO quantification in muscle lysates

Protein lysates from the previous dystrophin quantification experiments by IB and MS were used for PMO quantification by a high-sensitivity hybridization ELISA, as previously described [42]. In brief, sample lysates were diluted 1/20, 1/200, and 1/2000 in a control muscle lysate buffer (0.2ug/μL protein), and PMO standards were diluted to various concentrations in a similar manner. Hybridization was facilitated using an anti-sense probe to the PMO with both a biotin epitope and a DIG tag for hybridization and carried out at 37 °C. After hybridization, 100 μL of the hybridization mix was pipetted into duplicate wells on avidin-coated plates and incubated at 37 °C for 30 min. The plates were then washed, and each well was treated with micrococcal nuclease (NEB, M02475), followed by incubation with an anti-digoxigenin-AP Fab fragment antibody (Roche, 11093274910). Lastly, AttoPhos Substrate (Promega, S101C) was added to the plates and incubated at 37 °C for 30 min. Fluorescent readings were obtained, and PMO concentrations were quantified and calculated on the basis of the PMO standard curve.

### Statistical analysis

All data are presented as dystrophin percent of C57BL/10 (normal) and means ± standard deviation of the mean. Correlation analysis between quantification methods was performed to determine Spearman’s statistical correlations. *p* < 0.05 was considered significant.

## Results

### Dystrophin rescue is highly variable after a single 800 mg/kg IV injection

In order to fully characterize the level of dystrophin restored by exon skipping 1 month after a single high-dose PMO injection in *mdx* mice, we analyzed six different muscles from six animals by immunofluorescent (IF) staining and immunoblotting (IB). Also, the protein extracts that were prepared for IB from three muscles were analyzed using a quantitative mass spectrometry (MS) stable isotope spike-in approach. For each muscle, dystrophin expression was calculated as a percentage of normal as compared to C57BL/10 dystrophin protein levels. Table 1 shows the average dystrophin protein expression determined for each muscle group across all animals and the standard deviation for all three quantification methods. Expression levels of de novo dystrophin varied highly between animals and between muscles, regardless of the detection method used, ranging from essentially zero dystrophin up to 80 % of wild-type control levels (see Additional file 2 for individual values).

**Table 1:** Dystrophin quantification (% relative to WT)

Muscle	Immunofluorescence		Immunoblotting		Mass spectrometry	
	Mean ± S.D.	RSD %	Mean ± S.D.	RSD %	Mean ± S.D.	RSD %
Triceps	38.54 ± 27.45	71.0	27.19 ± 29.56	108.7	22.85 ± 29.69	130.0
Quadriceps	10.81 ± 10.64	98.3	2.46 ± 2.03	82.7	–	–
Diaphragm	10.6 ± 5.41	50.7	14.54 ± 10.40	71.6	–	–
Gastrocnemius	10.42 ± 7.87	75.5	8.36 ± 9.54	114.1	2.75 ± 2.40	87.1
Tibialis Anterior	8.70 ± 8.91	102.4	9.18 ± 12.88	140.3	9.12 ± 9.09	99.6
Heart	0.24 ± 0.22	93.2	0.49 ± 0.02	5.1	–	–

**Fig. 1:**
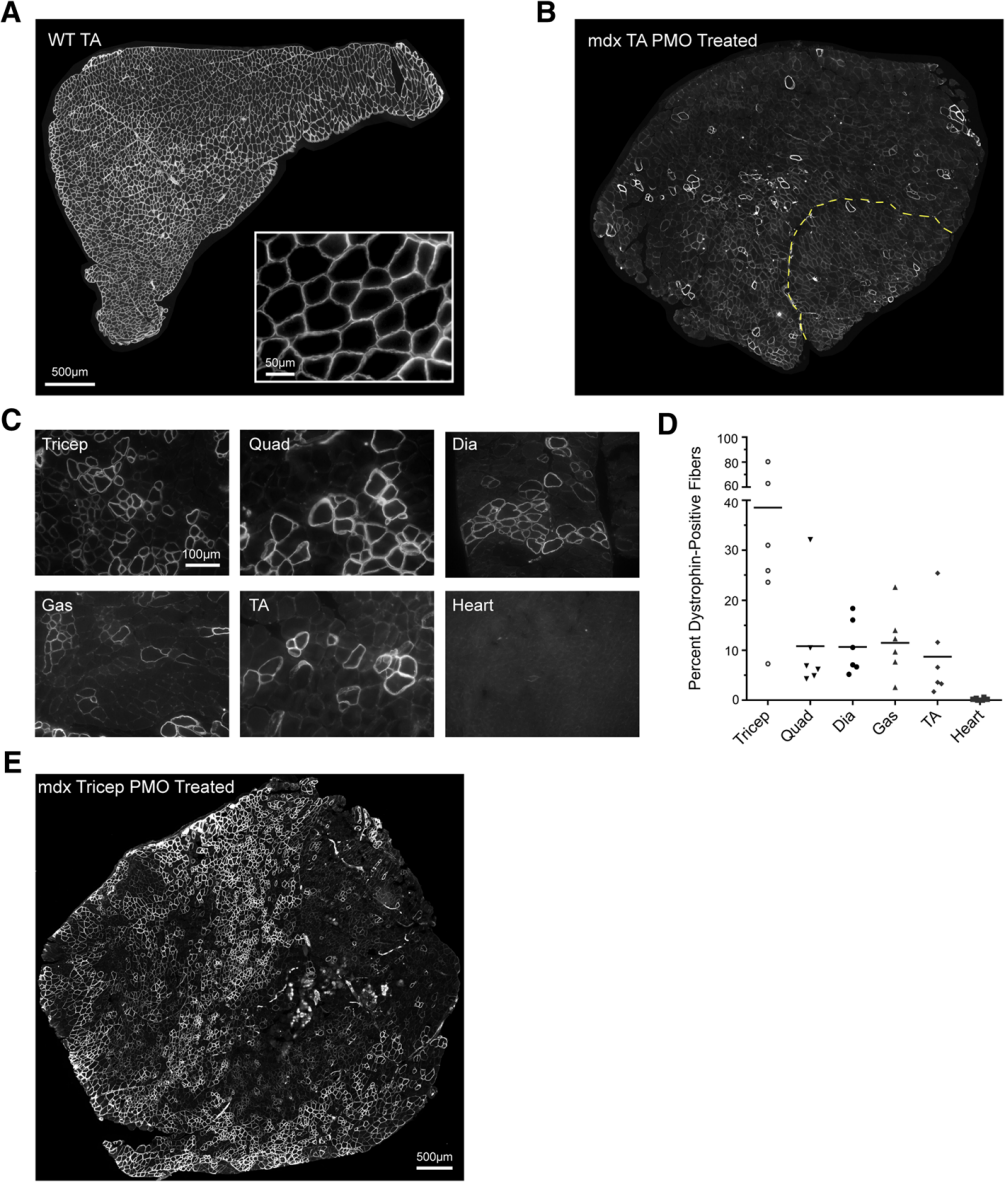
Variability of dystrophin protein expression, as shown by IF after PMO injection. **a** Representative images of C57BL/10 (WT) and **b** PMO-treated *mdx* tibialis anterior sections stained for dystrophin. The WT control shows uniform IF staining for dystrophin. Insert at ×40 shows expected staining pattern for dystrophin-positive fibers. **b** PMO-treated *mdx* tibialis anterior shows a mosaic staining pattern and clustering of positive fibers. The *yellow line* represents the border between the tibialis anterior and EDL. Quantification was performed on the entire area of the muscle section. **c** Representative images of mouse mdx-6 showing variability between the muscles of the same animal. Images were selected to show positive fiber clustering and do not represent total area quantification. **d** IF quantification of diaphragm, gastrocnemius, heart, quadriceps, tibialis anterior, and triceps for all mice (*n* = 6). **e** Geographic variability observed within the highly rescued triceps from mouse mdx-1. All tissues were sectioned (10-μm thick), stained, and probed with goat anti-rabbit IgG Alexa 594 antibody. Dystrophin-positive fibers were normalized to the area of the muscle section and the WT percentage of positive fibers. Original magnification for **a, b, e** = ×20; *scale bar*, 500 μm; for **c** = ×40; *scale bar*, 100 μm

Dystrophin protein was visualized by IF using anti-P7 antibody (Fig. 1a). A single high dose of PMO resulted in dystrophin localization to the membrane, which was detected with high geographic variability across the section (Fig. 1b, c). We found dystrophin-positive fibers to be clustered in groups throughout the section area, with positive and negative fibers interspersed (Fig. 1e). Muscle sections with low numbers of dystrophin-positive fibers, such as the quadriceps, also showed positive fibers isolated in small clusters, with the majority of the section area showing dystrophin-negative fibers (Fig. 1c).

The triceps muscle showed the highest dystrophin rescue levels, at an average of 38 % of normal (range 9 to 78 %, *n* = 6). We did not observe significant dystrophin-positive staining in cardiac muscle, other than sporadic positive fibers that may have been revertants (Fig. 1c). The other muscles evaluated averaged ~10 % of wild-type dystrophin levels (Fig. 1d).

The tibialis anterior muscle showed the highest level of variability among the six mice tested, with an average (mean ± SD) of 8.7 % ±8.91 % (a relative standard deviation of 102 %). The lowest variability was observed in the diaphragm muscle, with an average dystrophin level of 10.68 % ±5.41 % (a relative standard deviation of 50 %) (Table 1).

**Fig. 2:**
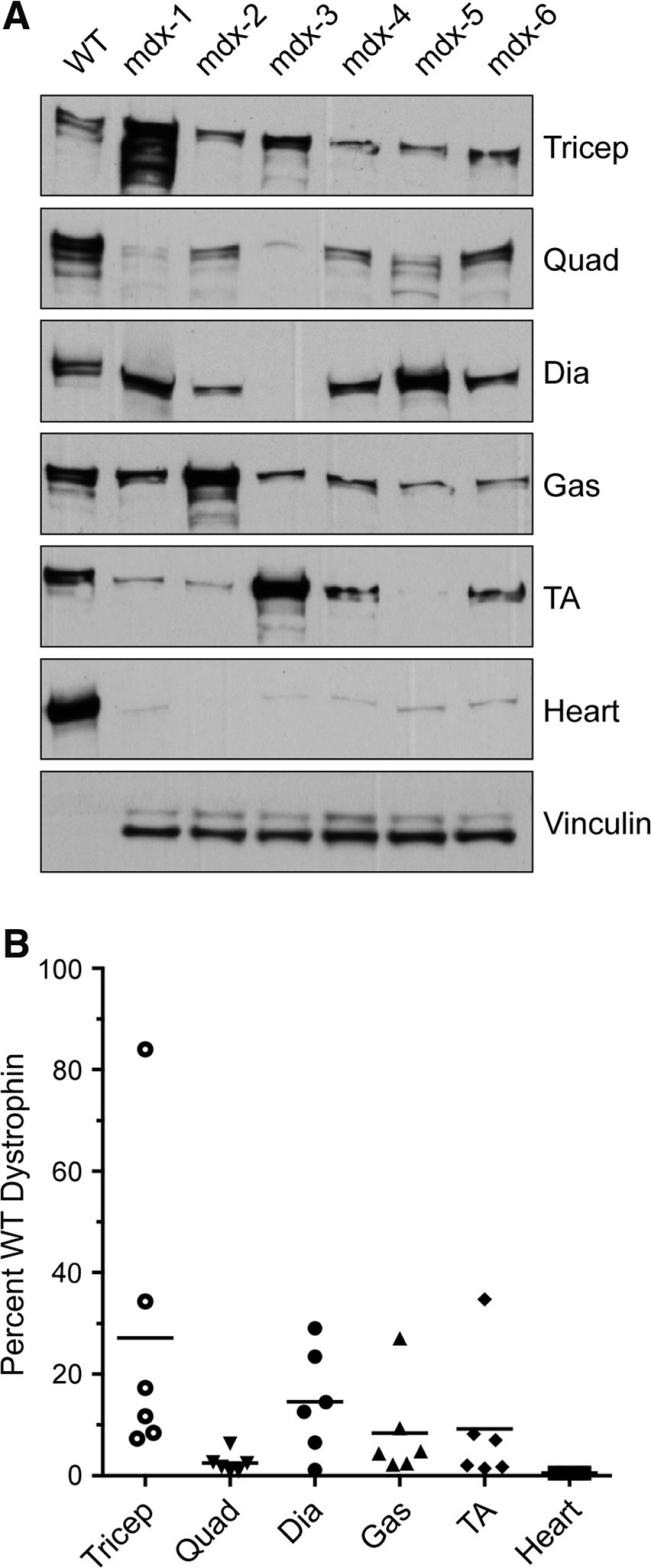
Dystrophin protein expression as detected by IB 1 month after PMO injection. **a** IB of protein lysates from diaphragm, heart, gastrocnemius, quadriceps, tibialis anterior, and triceps in PMO-treated *mdx* mice (*n* = 6) vs. WT control shows variability of dystrophin expression between muscles in PMO-injected *mdx* mice. There is variation in the same muscles between different mice (across) and different muscles in the same individual mouse (down). WT samples were serially diluted to 3.125 μg for protein loading. Vinculin (117 kDa) was used as a loading control. Densitometric analysis was performed using Quantity One software. **b** Dystrophin quantification by IB, demonstrating the percentage dystrophin expression in PMO-injected mice vs. WT (set to 100 %). *Plots* show high variability between mice within a muscle group and between muscles. All the data are presented as mean percentages

IB was carried out on muscle homogenate protein extracts to further quantify total dystrophin as a percentage of wild-type levels. Table 1 shows the average expression of dystrophin for each muscle analyzed, and Additional file 2 lists the individual sample values. Consistent with our IF results, IB-quantified dystrophin protein levels were highly variable between animals and muscle groups (Fig. 2a). We again observed dystrophin rescue to be the highest in the triceps (average 27 %, range 11 to 71 %) and the lowest in the quadriceps (average 2.5 %, range 1 to 9 %) (Fig. 2a, b). Notably, we found low levels of dystrophin protein in cardiac muscle 1 month after high-dose PMO injection (Table 1 and Fig. 2). Again, the tibialis anterior muscle showed the highest level of variability among the mice tested, with an average dystrophin level of 9.18 ± 12.88 % (a relative standard deviation of 140 %) (Table 1). It is important to note that the quantification dystrophin immunoblotting can be challenging due to potential proteolytic degradation of samples (Additional file 1B).

**Fig. 3:**
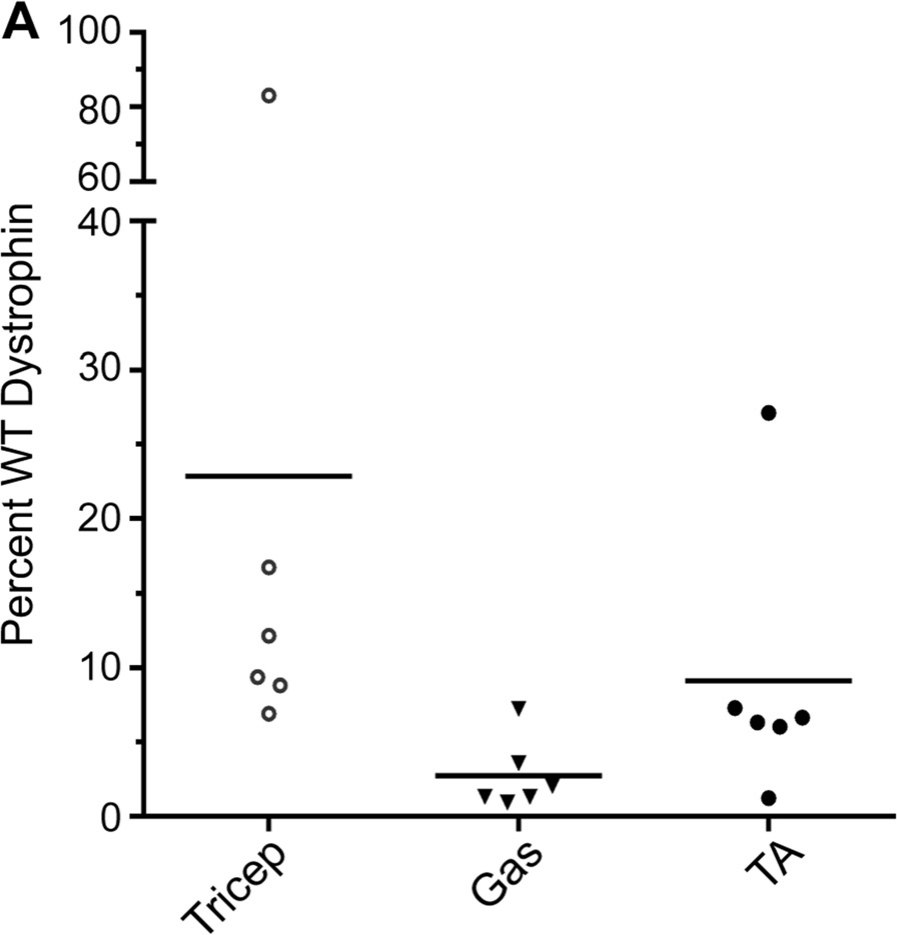
Dystrophin protein expression as detected by MS. **a** Triceps, tibialis anterior, and gastrocnemius RIPA buffer extracts were analyzed by MS. The percentage of dystrophin protein expression in PMO-injected *mdx* mice was compared to the dystrophin percentage in WT. All data are presented as mean percentages

We have recently reported on a mass spectrometry (MS) method to quantify dystrophin using stable isotope-labeled dystrophin peptides [39]. We performed quantitative MS on a subset of muscle protein homogenates that were used for IB. Dystrophin protein levels as quantified by MS were variable between the triceps, gastrocnemius, and tibialis anterior, consistent with previous results (Fig. 3). The triceps showed the highest average dystrophin expression (average 23 %, range 6 to 83 %). The tibialis anterior and gastrocnemius had lower levels (average 9 and 3 %, respectively), as shown in Table 1. The standard deviation range for MS also showed that dystrophin expression was highly variable between mice for a given muscle group (Table 1).

The results from all three quantification methods showed that dystrophin rescue was highly variable between animals and between the muscles tested; however, the triceps demonstrated the highest dystrophin expression levels overall (Table 1).

### Variation in de novo dystrophin restoration is independent of muscle/fiber type

Whole-muscle studies have indicated that adaptive changes in protein synthesis can occur in a myofiber type-dependent manner [43, 44]. To explore potential myofiber type differences in the rescue levels of the dystrophin protein, we compared muscles composed predominantly of fast-twitch fibers (EDL) to those composed of predominantly slow-twitch muscle (soleus) and also to muscles of mixed myofiber type (triceps).

**Fig. 4:**
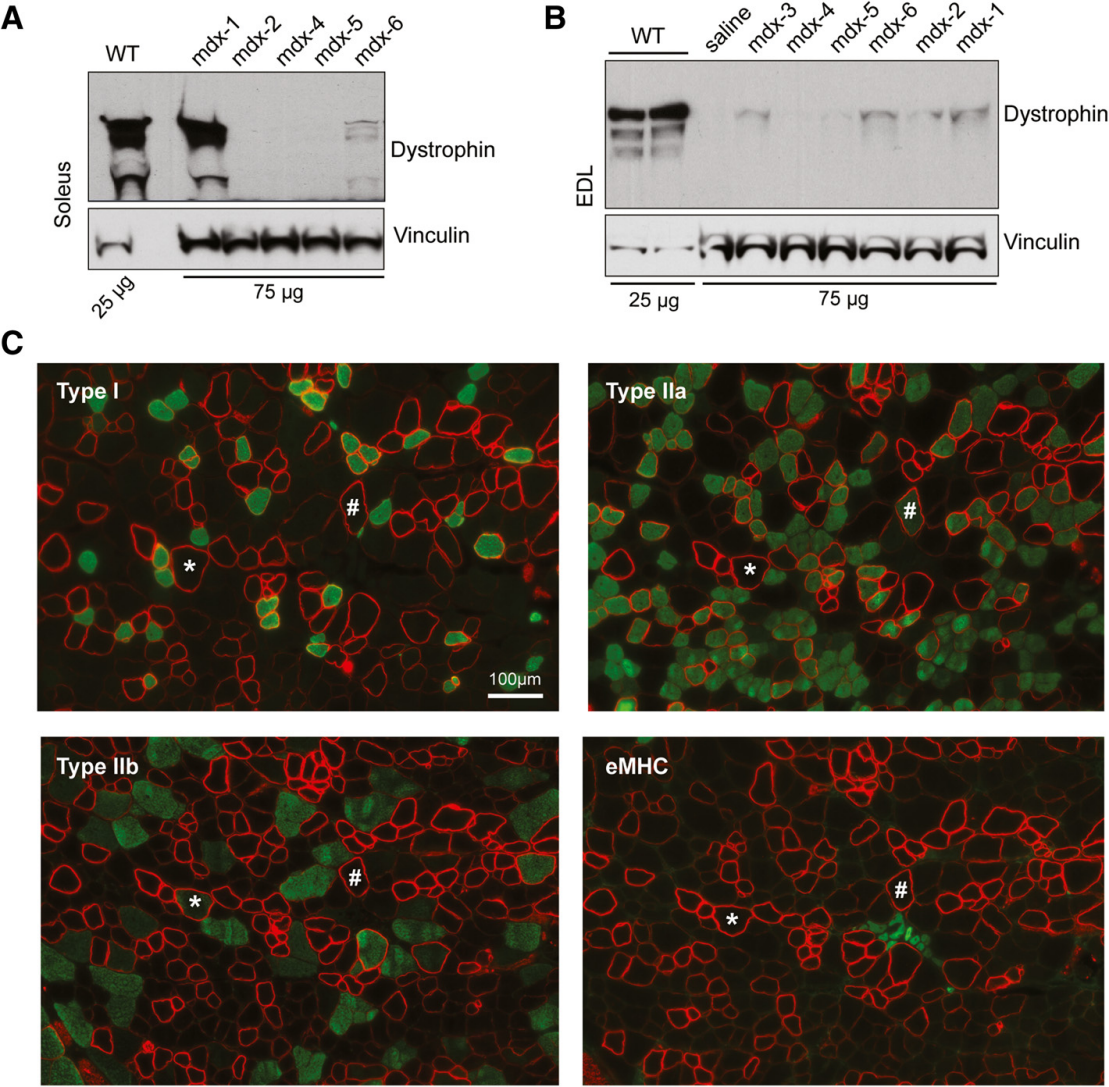
De novo dystrophin restoration varies independently of muscle/fiber type. **a** IB of the predominately slow-twitch muscle soleus and **b** fast-twitch EDL muscle shows no preference for dystrophin rescue by muscle fiber type. WT samples were loaded at 25 μg, and vinculin was used as a loading control. For *mdx* mice, 75 μg of total protein was loaded. **c** IF in serial sections from the triceps muscle that showed the highest dystrophin rescue for fiber-type identification of type 1, type 2a, type 2b, and embryonic myosin heavy chain (eMHC) isoforms (*green*) and dystrophin (*red*). *Asterisks* and *pound signs* indicate the same muscle fiber in different images. Original magnification for **a** = ×40; *scale bar*, 100 μm

We first blotted whole-muscle extracts to detect any potential preference of particular muscle types for dystrophin restoration upon exon skipping (Fig. 4a, b). By IB, we did not find any significant preferences in dystrophin restoration between the soleus (slow-twitch muscle) and EDL, a 100 % fast-twitch muscle. In fact, we observed the same variability pattern described above. For the soleus, one mouse (mdx-1) of the five tested showed very high dystrophin rescue, but low levels (or no protein) were detected in the other animals (Fig. 4a). For the EDL, low dystrophin levels were detected in all the mice but in variable amounts (Fig. 4b).

Although we saw no difference in the variability of dystrophin rescue between fast and slow muscles, we asked whether there might be a fiber-type preference for dystrophin rescue in the triceps, which contains mixed fiber populations. By IF, we identified four different fiber types (slow-twitch type 1, fast-twitch types 2a and 2b, and embryonic) and co-stained for dystrophin. We did not observe dystrophin rescue to be confined to a particular fiber type, since dystrophin-positive staining was seen in both the slow and fast fiber types in the triceps (Fig. 4c). The triceps contains a high proportion of type 2b fibers, with type 1 and type 2a fibers distributed in specific areas of the muscle. As demonstrated in Fig. 1, the dystrophin-positive fibers were clustered and geographically dispersed; large dystrophin-positive clusters were observed within regions rich in both slow (type 1)- and fast (type 2a and 2b)-twitch myofibers, suggesting that there is no fiber-type preference with regard to the efficiency of dystrophin rescue in the triceps (Additional file 3). We also saw a number of small regenerating fibers that were positive for the embryonic myosin heavy chain isoform, but we did not see any dystrophin expression in these fibers (Fig. 4c and Additional file 3). This result presumably reflects the fact that the new fibers were formed during the time period when PMO was no longer present.

### Dystrophin rescue does not correlate with residual morpholino concentration in tissue

To determine whether dystrophin rescue was correlated with residual morpholino in muscle, we measured PMO concentrations in the muscle lysate samples used for quantification of dystrophin by IB and MS. We hypothesized that highly rescued muscles would show better uptake and retention of PMO at 30 days after delivery. To investigate drug retention, morpholino concentrations were determined by a recently described high-sensitivity ELISA assay, which has a lower detection limit of 5 pM [42].

**Fig. 5:**
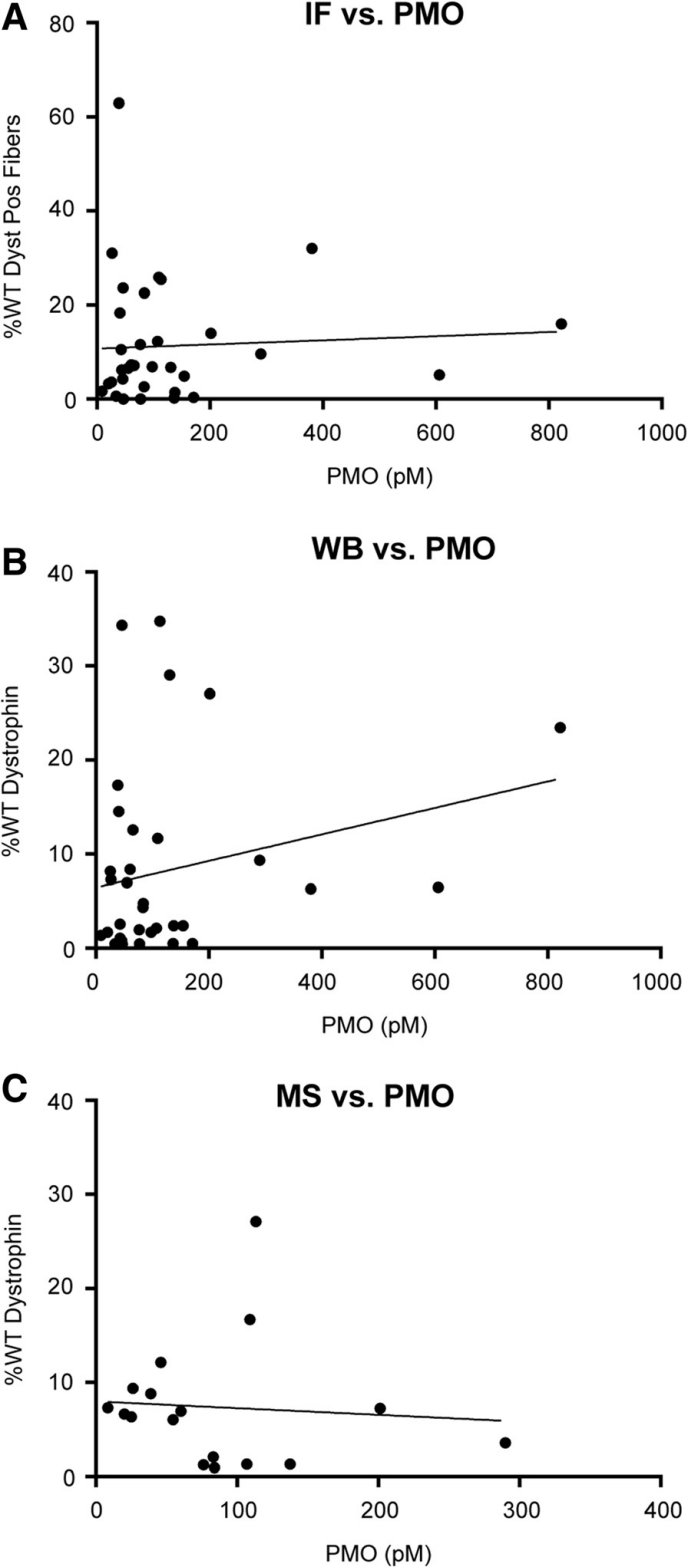
Correlation between residual PMO in muscle and de novo dystrophin. **a–c** Percentage of WT dystrophin as measured by **a** IF, and PMO levels measured by hybridization ELISA were plotted with a regression line. The same analysis was performed for dystrophin quantified by **b** IB and **c** MS. There was no significant correlation, as illustrated by the regression lines. Note that for MS we have fewer data points because we measured only three muscle groups by this method (*N* = 18)

After 30 days, we observed that residual PMO concentrations varied greatly among the muscles (Additional file 4) but saw no correlation between PMO concentration and de novo dystrophin levels after exon skipping, except for one outlier (Fig. 5). The high-responder mouse that showed over 80 % dystrophin rescue in the triceps by both IB and MS (mdx-1) showed a positive correlation with PMO retention at 1214.6 pM (Table 2). This data point skewed the correlation toward a positive trend and was then removed from further analysis (data not shown); without this sample, no significant correlation was found between residual PMO and dystrophin expression rescue (Fig. 5). Among all the muscles evaluated, the diaphragm showed the highest residual PMO concentration (822 pM ± 341 pM), and the tibialis anterior showed the lowest (49.6 ± 39.9 pM) (Table 2).

**Table 2:** Residual morpholino concentration 30 days after PMO administration

	PMO conc (pM/ug protein)					
ID	Tricep	Quad	Dia	Gas	TA	Heart
mdx-1	1214.6	380.2	822.1	289.9	76.2	171.0
mdx-2	109.1	97.7	606.1	201.2	20.2	46.3
mdx-3	45.8	154.1	42.6	82.9	113.3	77.1
mdx-4	60.1	45.2	40.1	83.8	25.0	33.4
mdx-5	26.2	42.7	130.4	106.7	8.4	45.1
mdx-6	38.8	43.4	65.3	137.4	54.5	136.6
Average	249.1	127.2	284.4	150.3	49.6	84.9
SD	473.9	131.5	341.3	81.4	39.9	56.3

**Fig. 6:**
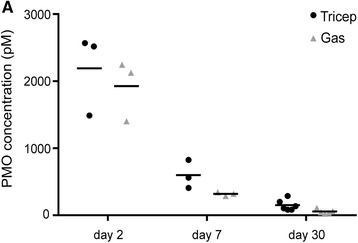
Time course of residual PMO concentration. **a** Residual morpholino concentration was measured in the triceps and gastrocnemius muscle extracts at 2 (*n* = 3), 7 (*n* = 3), and 30 (*n* = 6) days after PMO administration. No statistically significant differences were found between the concentrations of PMO in the two muscle groups for a given time point. We observed a decline in the PMO concentration in muscle over time

In addition, we also measured residual PMO concentrations at different time points to monitor PMO pharmacokinetics in muscle after a single high dose. Two additional groups (*n* = 3) were treated with PMO and sacrificed 2 and 7 days after delivery of a single high-bolus IV dose. We assessed PMO levels as a function of time in the triceps and gastrocnemius. The highest levels of PMO in muscle were quantified 2 days after PMO administration for both muscle groups. Subsequently, PMO levels decreased as a function of time (Fig. 6). Two days after PMO delivery, no dystrophin protein was detected by IB or IF (data not shown). On day 7, dystrophin protein was quantified at low levels by both IF and IB (Additional file 5). Our data suggest that dystrophin levels are detectable within a week of morpholino delivery and reach higher levels at day 30, although residual PMO concentrations are declining at that point.

## Discussion

Systemic delivery of AOs to dystrophin-deficient muscle is known to be dose-dependent, but variable effectiveness has been achieved in dystrophin correction (in mice, dogs, and humans). This variability in biochemical efficacy is generally seen between individual myofibers in the same muscle sample, as well as between different muscle groups (tested in dog and mouse), and between different individuals tested. We sought to test a number of variables that might underlie this variation, including residual drug in tissues (e.g., more residual drug may indicate greater success) and different muscle groups. An additional factor contributing to variability in the apparent success of exon skipping is the use of different methods to measure dystrophin. Both IF and IB have relatively low reliability in terms of quantifying low-abundance proteins, which presents a considerable problem in the context of exon skipping, for which only low dystrophin levels have been achieved with the current AOs in clinical trials (poor coefficients of variance (CVs)). Thus, the variability reported in exon skipping could be a consequence of unreliability in the quantification method. Here, we compared IF, IB, and stable isotope MS methods in the same samples. We found that all three dystrophin testing methods provided similar qualitative results, with a strongly positive muscle by one method also being strongly positive by the other two methods. Poor coefficients of variance were observed in muscles that had lower dystrophin rescue levels, highlighting the challenge of attempting to quantify low levels of dystrophin (Additional file 2). We also found a correlation between the transcript levels by real-time quantitative PCR (qPCR) and the dystrophin protein amounts in tibialis anterior muscle from the tested mice (Additional file 6).

We delivered a single high-dose IV injection (800 mg/kg) and examined muscles after 1 month. It is well established that repeated injections (generally weekly) increase the success of exon skipping; however, we felt that a single high-dose injection was technically more feasible and less variable, allowing for better interpretation of the results. Our data were consistent with previous reports, in which the amount of drug-induced dystrophin production was highly variable [18, 21]. We showed that the triceps muscle had the greatest degree of rescue (6–83 %), whereas other muscle groups showed generally lower rescue (3–9 %).

Despite considerable investigation, the molecular basis for variability in the level of dystrophin expression induced by AOs is still unclear. The extent of dystrophin rescue does not appear to be influenced by fiber type, since similar expression patterns were seen in fast and slow muscles, and no preference for individual fiber type was observed within muscles with mixed fiber types. It is attractive to hypothesize that variability in dystrophin rescue is driven by success in drug delivery to that muscle, and this relationship may be reflected in the preferential retention of the AO drug in the most highly rescued muscles. However, we saw no correlation between residual morpholino concentration and dystrophin content as measured by IF, IB, or MS. This analysis does not rule out the possibility that highly expressing muscles may have had greater initial delivery of morpholino at the time of injection, may have restored the dystrophin, and then subsequently lost the morpholino.

To begin to test this possibility, we compared the morpholino content of triceps (generally high dystrophin rescue) and gastrocnemius (generally low dystrophin rescue) at 2 and 7 days after injection. There were no significant differences in the morpholino content of the triceps and gastrocnemius at any time point, arguing against morpholino delivery and/or retention as a major factor driving the success of the dystrophin rescue. IV injections were delivered via the retro-orbital sinus, and it is possible that the triceps received the highest dose of morpholino because of its relative proximity to the orbital sinus; however, the lack of correlation with tissue morpholino concentration at 2 days post-injection would seem to argue against this explanation. Other factors not investigated here could have influenced the higher dystrophin rescue in the triceps, such as muscle activity and/or muscle regeneration.

Given that neither muscle fiber type nor residual drug levels appeared to have a significant effect on the variability seen in dystrophin expression, we must speculate on the other variables that could contribute to the success of exon skipping that were not examined in the present study. It is evident from all of the muscles studied that dystrophin rescue occurs in a patchwork fashion, with dystrophin-positive fibers occurring in large clusters in geographically distinct regions rather than being randomly distributed throughout the muscle cross section. This pattern is interesting in the context of DMD pathology, which is characterized by specific regions of inflammation, degeneration, and regeneration within the muscle. Hence, drug uptake and subsequent dystrophin expression may be influenced by the microenvironment surrounding each individual fiber.

The variable histopathology associated with DMD is thought to arise as a result of asynchronous regeneration, which creates muscle microenvironments with varying degrees of pro-inflammatory and pro-fibrotic signaling [45]. A recent study by our group showed that muscle inflammation is linked to the production of TNF-alpha-induced microRNAs that target the dystrophin mRNA and inhibit dystrophin translation in Becker muscular dystrophy patients [33]. We have demonstrated an inverse correlation between the expression of dystrophin-targeting microRNAs and dystrophin rescue following AO administration to *mdx* muscles, highlighting the potential for these microRNAs to influence the success of exon skipping in DMD. Hence, we believe that the inflammatory muscle microenvironment is likely to be at least partially responsible for the “patchwork” pattern of variability in the AO-induced dystrophin rescue we report here. Finally, based on these findings, it appears that co-administration of compounds such as prednisone to inhibit the miRNA-inducing aspect of inflammation, together with AOs, has considerable potential as a strategy for both improving the level of dystrophin rescue and for decreasing inter-patient variability in future clinical trials of these exon skipping drugs.

## Conclusions

Variation in the level of dystrophin expression induced by AOs remains a major problem in human trials. Here, we show that dystrophin rescue occurs in a sporadic patchy pattern which, interestingly, reflects the patchy nature of DMD/*mdx* pathology, characterized by discrete regions of inflammation, degeneration, and regeneration within a muscle. Our data from the *mdx* mouse demonstrate no correlation, at the microscopic level, of dystrophin induction by this means with individual anatomical muscle or with fiber types within muscle. Nor is there any correlation between residual drug concentration and dystrophin induction within whole muscles. We suggest therefore that other factors may play important parts success of exon skipping.

This highlights the challenges associated with quantifying dystrophin in clinical trials where the single small muscle biopsy taken from a DMD patient is unlikely to be representative of the whole musculature over which any therapeutic effect is distributed. Thus, while dystrophin quantification is an important part of assessment, the large inter-sample variability should be taken into consideration when interpreting the data in human clinical trials.

## Additional files

Additional file 1:
**Proteolysis in dystrophin immunoblotting and band quantification.** A) Dystrophin immunoblotting of six muscles (triceps, quadriceps, diaphragm, gastrocnemius, tibialis anterior, and heart). The panel shows the entire blot area of the cropped images shown in Fig. 2a. Mouse ear-tag IDs are shown on the gastrocnemius blot. B) For dystrophin band quantification, x-ray films were scanned, and densitometry analysis was carried out using a Bio-Rad GS-800 calibrated densitometer and Quantity One software. Band area to be quantified was determined by delineating the largest band, which was kept constant for quantification of all other lanes. Degradation products were not included in the quantification as shown. (PDF 128 kb) Additional file 2:
**Intra-sample variability—dystrophin protein (% relative to WT).** The table provides individual values for dystrophin quantification by IF and IB. Expression levels of de novo dystrophin varied highly between animals and between muscles, regardless of the detection method used. (PDF 365 kb) Additional file 3:
**Dystrophin rescue is not preferential to a particular myofiber type.** IF in serial sections were stained for fiber-type identification of type 1, type 2a, type 2b, and embryonic myosin heavy chain isoforms (green) and dystrophin (red). Top panel from triceps muscle with the highest level of dystrophin rescue at 78 % (mdx-1) shows no myofiber type 1 double staining, some type 2a, and most type 2b positive for dystrophin. Bottom panel is a triceps with lower dystrophin rescue at 27 % (mdx-3) showing the opposite distribution of double stain. Here, exon skipping took place in regions rich for type 1 and type 2a, but not type 2b. Embryonic myofibers were not observed on the high responder, while the region of embryonic fibers on the bottom panel was negative for dystrophin. Original magnification for A = ×40; scale bar 500 μm. (PDF 233 kb) Additional file 4:
**Residual PMO concentration by mouse and muscle group.** Residual morpholino concentration was measured in diaphragm, heart, gastrocnemius, quadriceps, tibialis anterior, and triceps muscle extracts at 30 days after PMO administration (*n* = 6). We observed variable retention levels of PMO between muscle groups and animals after 30 days of delivery. (PDF 119 kb) Additional file 5:
**Dystrophin protein expression detected by IF and WB after 7 days of PMO delivery.** Four mice were treated with one high dose of PMO (800 mg/kg) and sacrificed at 7 days. Tibialis anterior muscles were dissected and analyzed by IF and WB. We observed low levels of dystrophin protein by both quantification methods as compared to saline-treated mice. (PDF 265 kb) Additional file 6:
**Correlation between dystrophin transcript levels by real-time qPCR and dystrophin protein amounts in TA muscle.** We compared dystrophin protein levels by IF and WB with mRNA transcript levels at 30 days after one high-dose PMO injection (800 mg/kg). A) By RTqPCR, mdx-3 mouse shows the highest percent exon skipping, which translates to high dystrophin protein amounts as observed by B) WB and C) IF. (PDF 158 kb)

## Abbreviations

AOs: anti-sense oligonucleotides
DMD: Duchenne muscular dystrophy
EDL: extensor digitorum longus
eMHC: embryonic myosin heavy chain
IB: immunoblotting
IF: immunofluorescence
mRNA: messenger RNA
MS: mass spectrometry
PMO or morpholino: phosphorodiamidate morpholino oligomer
RSD: relative standard deviation
TA: tibialis anterior
Gas: gastrocnemius
Dia: diaphragm

## References

[1] Hoffman EP (1993). Genotype/phenotype correlations in Duchenne/Becker dystrophy. Mol Cell Biol Hum Dis Serv.

[2] Hoffman EP, Monaco AP, Feener CC, Kunkel LM (1987). Conservation of the Duchenne muscular dystrophy gene in mice and humans. Science.

[3] Hoffman EP, Schwartz L (1991). Dystrophin and disease. Mol Aspects Med.

[4] Roses AD, Herbstreith MH, Appel SH (1975). Membrane protein kinase alteration in Duchenne muscular dystrophy. Nature.

[5] Hutter OF, Burton FL, Bovell DL (1991). Mechanical properties of normal and mdx mouse sarcolemma: bearing on function of dystrophin. J Muscle Res Cell Motil.

[6] Menke A, Jockusch H (1991). Decreased osmotic stability of dystrophin-less muscle cells from the mdx mouse. Nature.

[7] Menke A, Jockusch H (1995). Extent of shock-induced membrane leakage in human and mouse myotubes depends on dystrophin. J Cell Sci.

[8] Pasternak C, Wong S, Elson EL (1995). Mechanical function of dystrophin in muscle cells. J Cell Biol.

[9] Emery AE (2002). The muscular dystrophies. Lancet.

[10] Gilroy J, Cahalan JL, Berman R, Newman M (1963). Cardiac and pulmonary complications in Duchenne’s progressive muscular dystrophy. Circulation.

[11] Melacini P, Vianello A, Villanova C, Fanin M, Miorin M, Angelini C (1996). Cardiac and respiratory involvement in advanced stage Duchenne muscular dystrophy. Neuromuscul Disord.

[12] Manzur AY, Kinali M, Muntoni F (2008). Update on the management of Duchenne muscular dystrophy. Arch Dis Child.

[13] Griggs RC, Herr BE, Reha A, Elfring G, Atkinson L, Cwik V (2013). Corticosteroids in Duchenne muscular dystrophy: major variations in practice. Muscle Nerve.

[14] Bushby K, Finkel R, Birnkrant DJ, Case LE, Clemens PR, Cripe L (2010). Diagnosis and management of Duchenne muscular dystrophy, part 2: implementation of multidisciplinary care. Lancet Neurol.

[15] Bushby K, Finkel R, Birnkrant DJ, Case LE, Clemens PR, Cripe L (2010). Diagnosis and management of Duchenne muscular dystrophy, part 1: diagnosis, and pharmacological and psychosocial management. Lancet Neurol.

[16] Wood MJ, Gait MJ, Yin H (2010). RNA-targeted splice-correction therapy for neuromuscular disease. Brain.

[17] Lu QL, Rabinowitz A, Chen YC, Yokota T, Yin H, Alter J (2005). Systemic delivery of antisense oligoribonucleotide restores dystrophin expression in body-wide skeletal muscles. Proc Natl Acad Sci U S A.

[18] Alter J, Lou F, Rabinowitz A, Yin H, Rosenfeld J, Wilton SD (2006). Systemic delivery of morpholino oligonucleotide restores dystrophin expression bodywide and improves dystrophic pathology. Nat Med.

[19] Yokota T, Lu QL, Partridge T, Kobayashi M, Nakamura A, Takeda S (2009). Efficacy of systemic morpholino exon-skipping in Duchenne dystrophy dogs. Ann Neurol.

[20] Cirak S, Arechavala-Gomeza V, Guglieri M, Feng L, Torelli S, Anthony K (2011). Exon skipping and dystrophin restoration in patients with Duchenne muscular dystrophy after systemic phosphorodiamidate morpholino oligomer treatment: an open-label, phase 2, dose-escalation study. Lancet.

[21] Wu B, Lu P, Benrashid E, Malik S, Ashar J, Doran TJ (2010). Dose-dependent restoration of dystrophin expression in cardiac muscle of dystrophic mice by systemically delivered morpholino. Gene Ther.

[22] Aoki Y, Nakamura A, Yokota T, Saito T, Okazawa H, Nagata T (2010). In-frame dystrophin following exon 51-skipping improves muscle pathology and function in the exon 52-deficient mdx mouse. Mol Ther.

[23] Malerba A, Sharp PS, Graham IR, Arechavala-Gomeza V, Foster K, Muntoni F (2011). Chronic systemic therapy with low-dose morpholino oligomers ameliorates the pathology and normalizes locomotor behavior in mdx mice. Mol Ther.

[24] Kinali M, Arechavala-Gomeza V, Feng L, Cirak S, Hunt D, Adkin C (2009). Local restoration of dystrophin expression with the morpholino oligomer AVI-4658 in Duchenne muscular dystrophy: a single-blind, placebo-controlled, dose-escalation, proof-of-concept study. Lancet Neurol.

[25] Mendell JR, Rodino-Klapac LR, Sahenk Z, Roush K, Bird L, Lowes LP (2013). Eteplirsen for the treatment of Duchenne muscular dystrophy. Ann Neurol.

[26] Goemans NM, Tulinius M, van den Akker JT, Burm BE, Ekhart PF, Heuvelmans N (2011). Systemic administration of PRO051 in Duchenne’s muscular dystrophy. N Engl J Med.

[27] Voit T, Topaloglu H, Straub V, Muntoni F, Deconinck N, Campion G (2014). Safety and efficacy of drisapersen for the treatment of Duchenne muscular dystrophy (DEMAND II): an exploratory, randomised, placebo-controlled phase 2 study. Lancet Neurol.

[28] Yokota T, Pistilli E, Duddy W, Nagaraju K (2007). Potential of oligonucleotide-mediated exon-skipping therapy for Duchenne muscular dystrophy. Expert Opin Biol Ther.

[29] Sharp PS, Bye-a-Jee H, Wells DJ (2011). Physiological characterization of muscle strength with variable levels of dystrophin restoration in mdx mice following local antisense therapy. Mol Ther.

[30] Sazani P, Van Ness KP, Weller DL, Poage D, Nelson K, Shrewsbury SB (2011). Chemical and mechanistic toxicology evaluation of exon skipping phosphorodiamidate morpholino oligomers in mdx mice. Int J Toxicol.

[31] Hoffman EP, Bronson A, Levin AA, Takeda S, Yokota T, Baudy AR (2011). Restoring dystrophin expression in Duchenne muscular dystrophy muscle progress in exon skipping and stop codon read through. Am J Pathol.

[32] Hoffman EP, McNally EM (2014). Exon-skipping therapy: a roadblock, detour, or bump in the road. Sci Transl Med.

[33] Fiorillo AA, Heier CR, Novak JS, Tully CB, Brown KJ, Uaesoontrachoon K (2015). TNF-alpha-Induced microRNAs control dystrophin expression in Becker muscular dystrophy. Cell Rep.

[34] Baudy AR, Sali A, Jordan S, Kesari A, Johnston HK, Hoffman EP (2011). Non-invasive optical imaging of muscle pathology in mdx mice using cathepsin caged near-infrared imaging. Mol Imaging Biol.

[35] Zhang A (2015). The use of urinary and kidney SILAM proteomics to monitor kidney response to high dose morpholino oligonucleotides in the mdx mouse. Toxic Rep.

[36] Spurney CF, Gordish-Dressman H, Guerron AD, Sali A, Pandey GS, Rawat R (2009). Preclinical drug trials in the mdx mouse: assessment of reliable and sensitive outcome measures. Muscle Nerve.

[37] Kesari A, Fukuda M, Knoblach S, Bashir R, Nader GA, Rao D (2008). Dysferlin deficiency shows compensatory induction of Rab27A/Slp2a that may contribute to inflammatory onset. Am J Pathol.

[38] Goodman CA, Kotecki JA, Jacobs BL, Hornberger TA (2012). Muscle fiber type-dependent differences in the regulation of protein synthesis. PLoS ONE.

[39] Brown KJ, Marathi R, Fiorillo AA, Ciccimaro EF, Sharma S, Rowlands DS et al. Accurate quantitation of dystrophin protein in human skeletal muscle using mass spectrometry. J Bioanal Biomed. 2012;Suppl 7. 10.4172/1948-593X.S7-001 PMC3642779 23646235

[40] Rayavarapu S, Coley W, Cakir E, Jahnke V, Takeda S, Aoki Y (2013). Identification of disease specific pathways using in vivo SILAC proteomics in dystrophin deficient mdx mouse. Mol Cell Proteomics.

[41] Sharma N, Medikayala S, Defour A, Rayavarapu S, Brown KJ, Hathout Y (2012). Use of quantitative membrane proteomics identifies a novel role of mitochondria in healing injured muscles. J Biol Chem.

[42] Burki U, Keane J, Blain A, O’Donovan L, Gait MJ, Laval SH (2015). Development and application of an ultrasensitive hybridization-based ELISA method for the determination of peptide-conjugated phosphorodiamidate morpholino oligonucleotides. Nucleic Acid Ther.

[43] Mittendorfer B, Andersen JL, Plomgaard P, Saltin B, Babraj JA, Smith K (2005). Protein synthesis rates in human muscles: neither anatomical location nor fibre-type composition are major determinants. J Physiol.

[44] Savary I, Debras E, Dardevet D, Sornet C, Capitan P, Prugnaud J (1998). Effect of glucocorticoid excess on skeletal muscle and heart protein synthesis in adult and old rats. Br J Nutr.

[45] Dadgar S, Wang Z, Johnston H, Kesari A, Nagaraju K, Chen YW (2014). Asynchronous remodeling is a driver of failed regeneration in Duchenne muscular dystrophy. J Cell Biol.

