# Supplementary material for: Elusive sources of variability of dystrophin rescue by exon skipping
Source: Skelet Muscle. 2015 Dec 1;5:44. doi: 10.1186/s13395-015-0070-6 (PMC4667482; doi:10.1186/s13395-015-0070-6)
Supplement: Additional file 1: — Proteolysis in dystrophin immunoblotting and band quantification. A) Dystrophin immunoblotting of six muscles (triceps, quadriceps, diaphragm, gastrocnemius, tibialis anterior, and heart). The panel shows the entire blot area of the cropped images shown in Fig. 2a. Mouse ear-tag IDs are shown on the gastrocnemius blot. B) For dystrophin band quantification, x-ray films were scanned, and densitometry analysis was carried out using a Bio-Rad GS-800 calibrated densitometer and Quantity One software. Band area to be quantified was determined by delineating the largest band, which was kept constant for quantification of all other lanes. Degradation products were not included in the quantification as shown. (PDF 128 kb) [file 13395_2015_70_MOESM1_ESM.pdf]

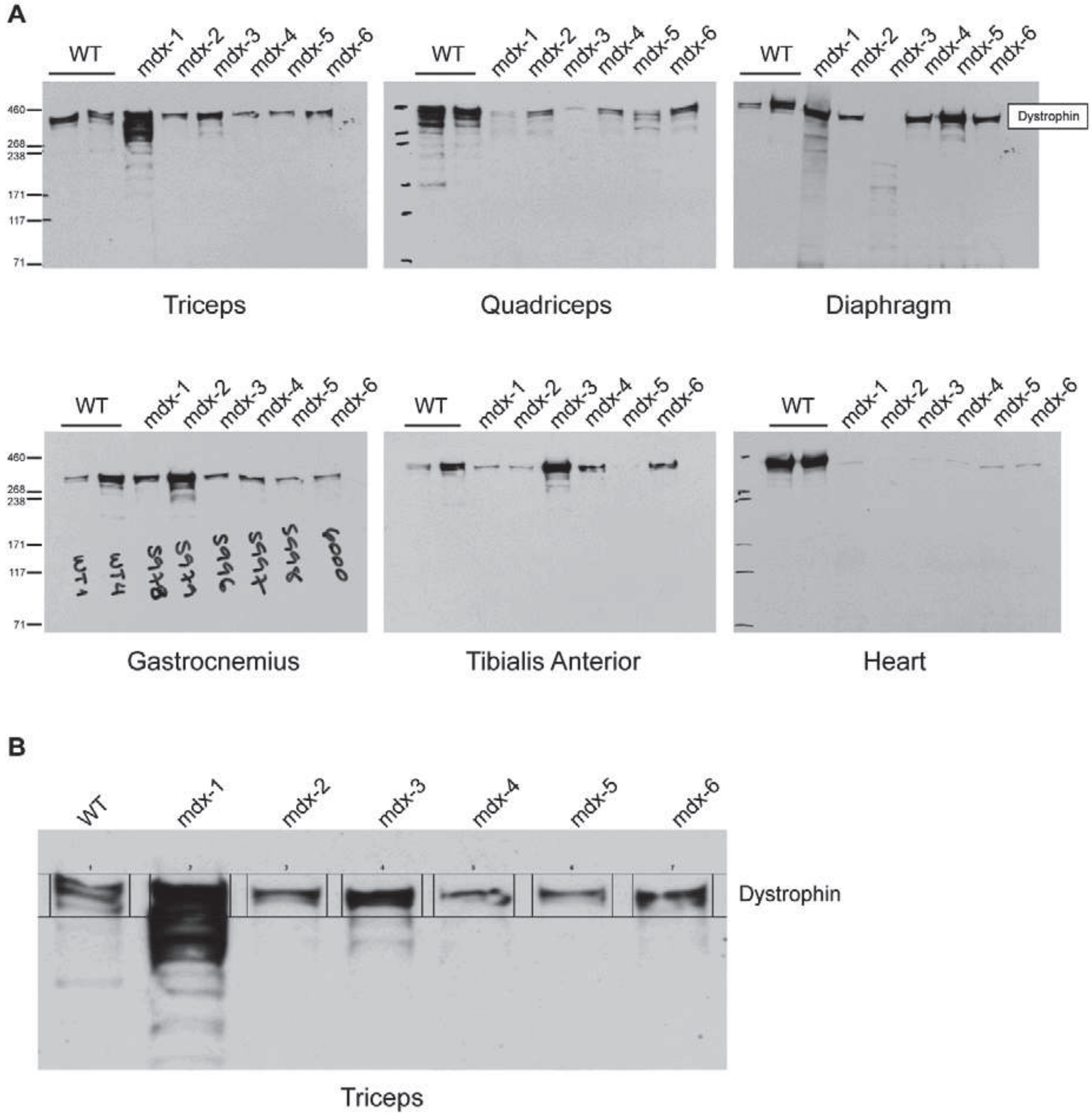

Additional File 1: Proteolysis in dystrophin immunoblotting and band quantification. A) Dystrophin immunoblotting of six muscles (triceps, quadriceps, diaphragm, gastrocnemius, tibialis anterior and heart). The panel shows the entire blot area of the cropped images shown in Figure 2A. Mouse ear-tag IDs are shown on the gastrocnemius blot. B) For dystrophin band quantification, x-ray films were scanned, and densitometry analysis was carried out using a BioRad GS-800 calibrated densitometer and Quantity One software. Band area to be quantified was determined by delineating the largest band, which was kept constant for quantification of all other lanes. Degradation products were not included in the quantification as shown.
