# Supplementary material for: Elusive sources of variability of dystrophin rescue by exon skipping
Source: Skelet Muscle. 2015 Dec 1;5:44. doi: 10.1186/s13395-015-0070-6 (PMC4667482; doi:10.1186/s13395-015-0070-6)
Supplement: Additional file 2: — Intra-sample variability—dystrophin protein (% relative to WT). The table provides individual values for dystrophin quantification by IF and IB. Expression levels of de novo dystrophin varied highly between animals and between muscles, regardless of the detection method used. (PDF 365 kb) [file 13395_2015_70_MOESM2_ESM.pdf]

	Immunofluorescence											
	Triceps		Quadriceps		Diaphragm		Gastroc		TA		Heart	
ID	Mean ± SD	CV%	Mean ± SD	CV%	Mean ± SD	CV%	Mean ± SD	CV%	Mean ± SD	CV%	Mean ± SD	CV%
mdx-1	78.34 ± 2.98	3.8	29.47 ± 2.68	12.5	15.34 ± 1.00	6.6	6.72 ± 4.15	61.8	12.66 ± 0.91	7.2	0.39 ± 0.02	4.0
mdx-2	33.65 ± 10.94	32.5	5.38 ± 2.19	40.8	4.52 ± 0.92	20.3	23.60 ± 13.57	57.5	6.70 ± 2.15	47.1	0.04 ± 0.002	5.2
mdx-3	26.88 ± 4.61	17.1	3.56 ± 1.85	52.0	8.50 ± 2.91	34.2	2.79 ± 0.25	8.8	39.54 ± 12.70	32.1	0.02 ± 0.003	12.9
mdx-4	8.92 ± 2.32	26.0	3.36 ± 1.34	39.8	14.92 ± 4.90	32.9	19.77 ± 4.00	20.2	6.82 ± 4.55	66.8	0.57 ± 0.06	11.1
mdx-5	22.49 ± 12.11	53.8	10.72 ± 0.28	2.6	5.27 ± 2.08	39.4	12.07 ± 0.37	3.1	3.42 ± 2.42	70.7	0.16 ± 0.01	7.0
mdx-6	61.06 ± 2.66	4.4	5.33 ± 1.23	23.1	5.83 ± 1.85	31.8	1.28 ± 0.25	19.2	6.68 ± 0.12	1.8	0.17 ± 0.07	38.9
Avg		22.9		28.5		27.5		28.4		37.6		13.2
	Western Blot											
	Triceps		Quadriceps		Diaphragm		Gastroc		TA		Heart	
ID	Mean ± SD	CV%	Mean ± SD	CV%	Mean ± SD	CV%	Mean ± SD	CV%	Mean ± SD	CV%	Mean ± SD	CV%
mdx-1	71.06 ± 18.34	25.8	9.55 ± 4.56	47.8	21.39 ± 2.97	13.9	10.09 ± 1.02	10.1	1.59 ± 0.59	37.0	0.78 ± 0.29	40.3
mdx-2	15.80 ± 5.82	36.9	6.21 ± 6.36	102.4	7.59 ± 1.57	20.7	25.27 ± 2.57	10.2	1.36 ± 0.51	37.7	0.56 ± 0.14	25.3
mdx-3	32.78 ± 2.23	6.8	7.34 ± 6.96	94.8	0.96 ± 0.17	0.18	4.54 ± 0.26	5.6	26.71 ± 11.42	42.8	0.59 ± 0.18	29.9
mdx-4	11.73 ± 10.36	65.9	1.81 ± 1.41	78.1	15.82 ± 1.81	11.5	4.16 ± 0.87	20.8	7.57 ± 0.88	11.6	0.76 ± 0.39	51.6
mdx-5	11.48 ± 5.87	51.1	8.81 ± 8.81	100.0	25.56 ± 3.54	13.3	2.73 ± 0.82	30.0	1.10 ± 0.39	35.5	0.76 ± 0.36	47.0
mdx-6	23.96 ± 9.35	39.0	1.33 ± 0.61	46.0	13.03 ± 0.65	5.0	2.30 ± 0.15	6.5	7.19 ± 0.30	4.1	0.75 ± 0.34	44.8
Avg		37.6		78.2		13.8		13.9		28.1		39.8

Additional File 2: Intra-sample variability – Dystrophin protein (% relative to WT). The table provides individual values for dystrophin quantification by IF and IB. Expression levels of *de novo* dystrophin varied highly between animals and between muscles, regardless of the detection method used.
