# Supplementary material for: Elusive sources of variability of dystrophin rescue by exon skipping
Source: Skelet Muscle. 2015 Dec 1;5:44. doi: 10.1186/s13395-015-0070-6 (PMC4667482; doi:10.1186/s13395-015-0070-6)
Supplement: Additional file 3: — Dystrophin rescue is not preferential to a particular myofiber type. IF in serial sections were stained for fiber-type identification of type 1, type 2a, type 2b, and embryonic myosin heavy chain isoforms (green) and dystrophin (red). Top panel from triceps muscle with the highest level of dystrophin rescue at 78 % (mdx-1) shows no myofiber type 1 double staining, some type 2a, and most type 2b positive for dystrophin. Bottom panel is a triceps with lower dystrophin rescue at 27 % (mdx-3) showing the opposite distribution of double stain. Here, exon skipping took place in regions rich for type 1 and type 2a, but not type 2b. Embryonic myofibers were not observed on the high responder, while the region of embryonic fibers on the bottom panel was negative for dystrophin. Original magnification for A = ×40; scale bar 500 μm. (PDF 233 kb) [file 13395_2015_70_MOESM3_ESM.pdf]

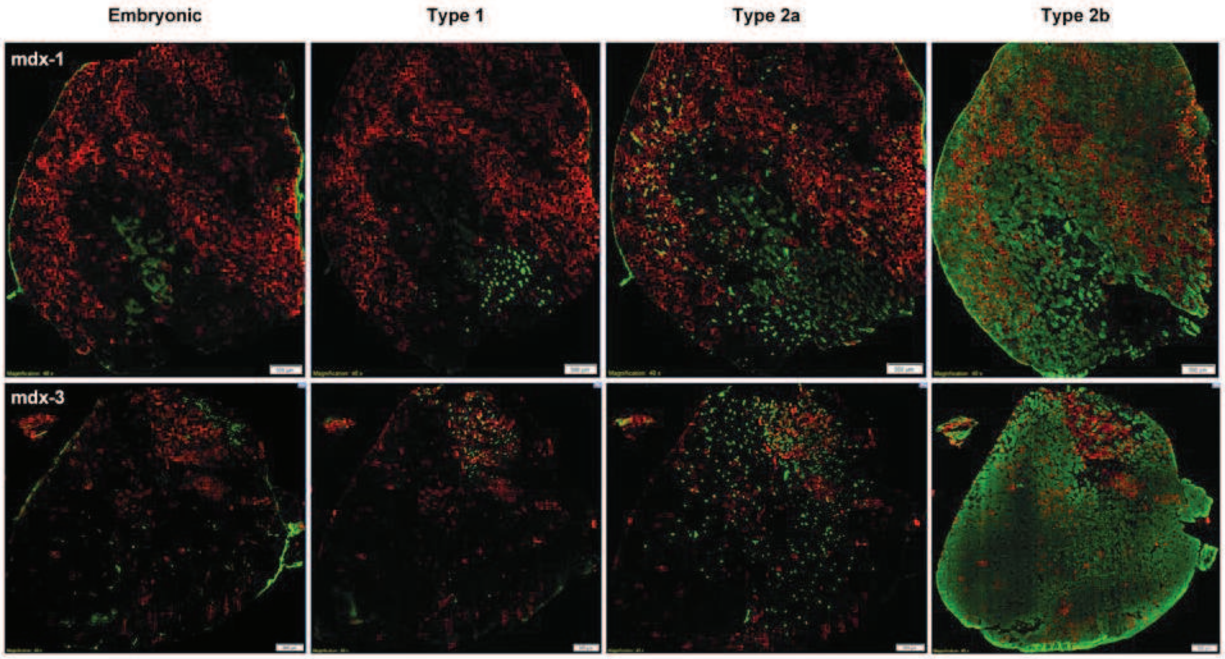

Additional File 3: Dystrophin rescue is not preferential to a particular myofiber type. IF in serial sections were stained for fiber type identification of type 1, type 2a, type 2b, and embryonic myosin heavy chain isoforms (green) and dystrophin (red). Top panel from triceps muscle with the highest level of dystrophin rescue at 78% (mdx-1) shows no myofiber type 1 double staining, some type 2a and most type 2b positive for dystrophin. Bottom panel is a triceps with lower dystrophin rescue at 27% (mdx-3) showing the opposite distribution of double stain. Here, exon skipping took place in regions rich for type 1 and type 2a, but not type 2b. Embryonic myofibers were not observed on the high responder, while the region of embryonic fibers on the bottom panel was negative for dystrophin. Original magnification for A = x40; scale bar 500µm.
