# Supplementary material for: Elusive sources of variability of dystrophin rescue by exon skipping
Source: Skelet Muscle. 2015 Dec 1;5:44. doi: 10.1186/s13395-015-0070-6 (PMC4667482; doi:10.1186/s13395-015-0070-6)
Supplement: Additional file 4: — Residual PMO concentration by mouse and muscle group. Residual morpholino concentration was measured in diaphragm, heart, gastrocnemius, quadriceps, tibialis anterior, and triceps muscle extracts at 30 days after PMO administration (n = 6). We observed variable retention levels of PMO between muscle groups and animals after 30 days of delivery. (PDF 119 kb) [file 13395_2015_70_MOESM4_ESM.pdf]

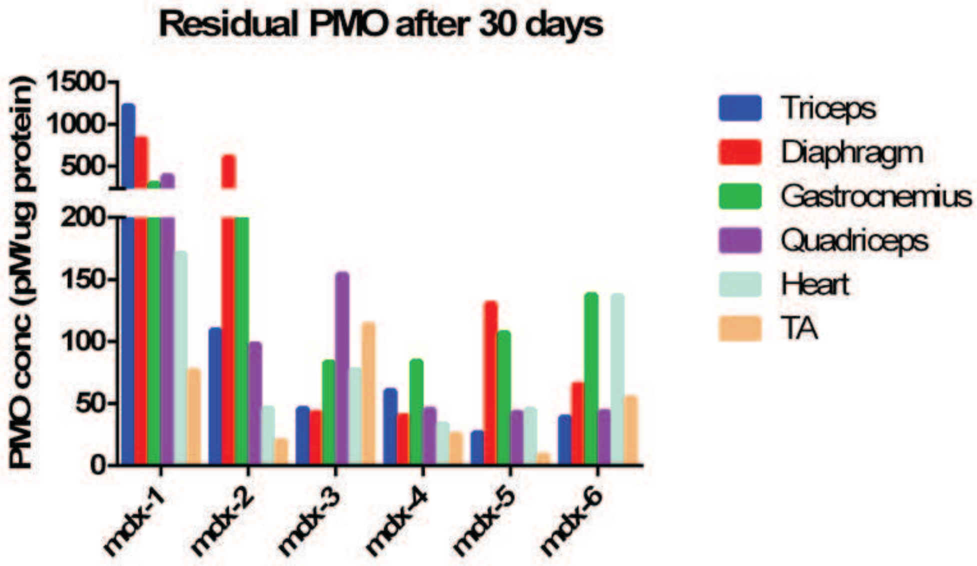

Additional File 4: Residual PMO concentration by mouse and muscle group. Residual morpholino concentration was measured in diaphragm, heart, gastrocnemius, quadriceps, tibialis anterior and triceps muscle extracts at 30 days after PMO administration (n=6). We observed variable retention levels of PMO between muscle groups and animals after 30 days of delivery.
