# Supplementary material for: Elusive sources of variability of dystrophin rescue by exon skipping
Source: Skelet Muscle. 2015 Dec 1;5:44. doi: 10.1186/s13395-015-0070-6 (PMC4667482; doi:10.1186/s13395-015-0070-6)
Supplement: Additional file 5: — Dystrophin protein expression detected by IF and WB after 7 days of PMO delivery. Four mice were treated with one high dose of PMO (800 mg/kg) and sacrificed at 7 days. Tibialis anterior muscles were dissected and analyzed by IF and WB. We observed low levels of dystrophin protein by both quantification methods as compared to saline-treated mice. (PDF 265 kb) [file 13395_2015_70_MOESM5_ESM.pdf]

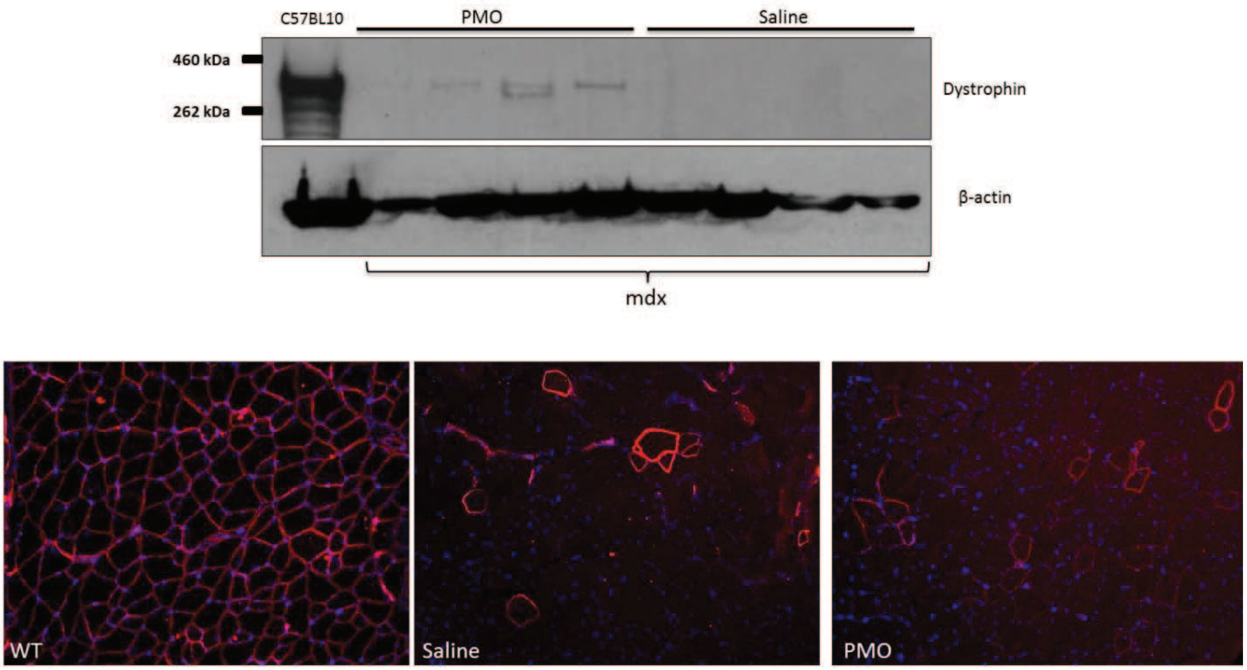

Additional File 5: Dystrophin protein expression detected by IF and WB after 7 days of PMO delivery. Four mice were treated with one high dose of PMO (800mg/kg) and sacrificed at seven days. Tibialis anterior muscles were dissected and analyzed by IF and WB. We observed low levels of dystrophin protein by both quantification methods as compared to saline treated mice.
