# Supplementary material for: Elusive sources of variability of dystrophin rescue by exon skipping
Source: Skelet Muscle. 2015 Dec 1;5:44. doi: 10.1186/s13395-015-0070-6 (PMC4667482; doi:10.1186/s13395-015-0070-6)
Supplement: Additional file 6: — Correlation between dystrophin transcript levels by real-time qPCR and dystrophin protein amounts in TA muscle. We compared dystrophin protein levels by IF and WB with mRNA transcript levels at 30 days after one high-dose PMO injection (800 mg/kg). A) By RTqPCR, mdx-3 mouse shows the highest percent exon skipping, which translates to high dystrophin protein amounts as observed by B) WB and C) IF. (PDF 158 kb) [file 13395_2015_70_MOESM6_ESM.pdf]

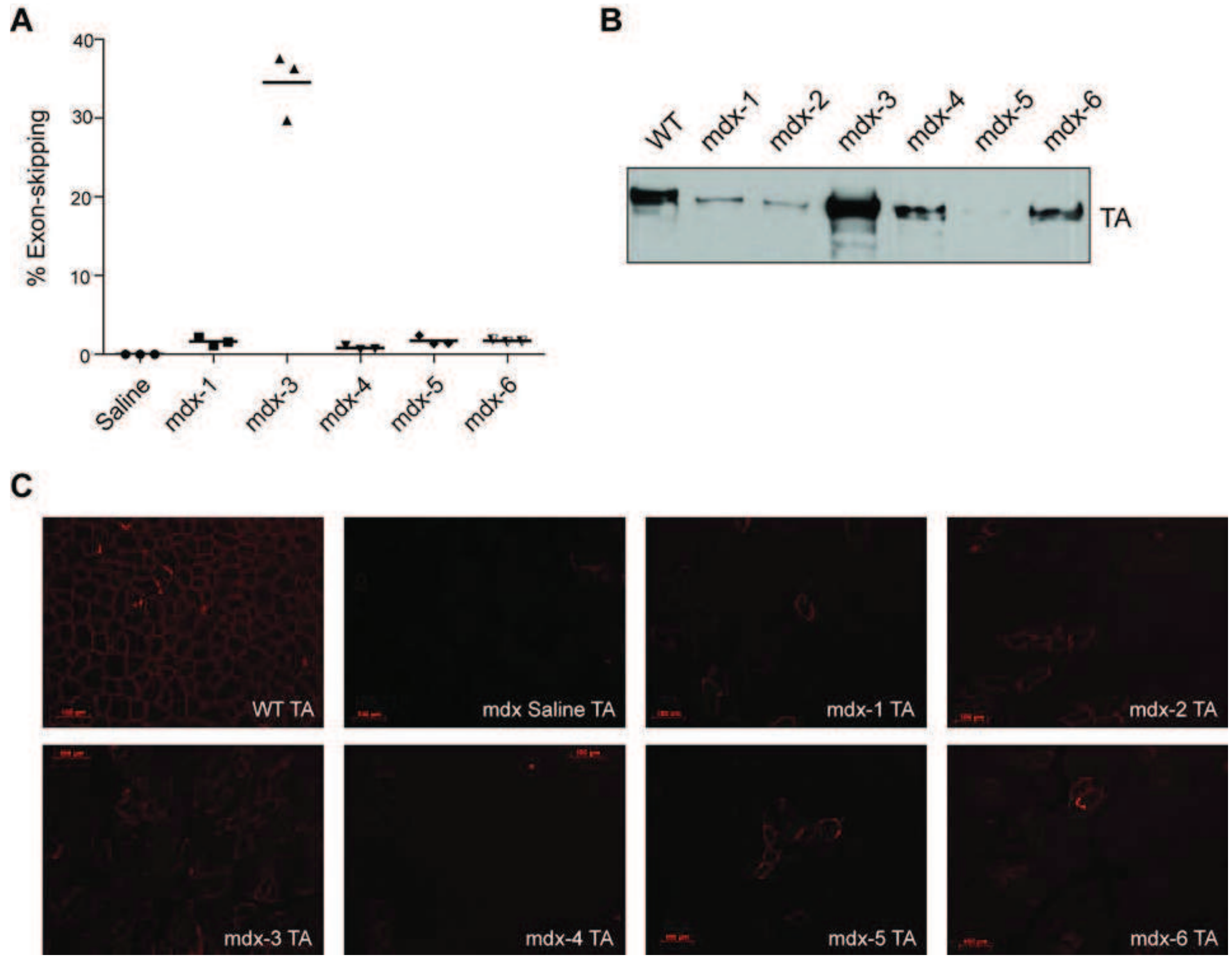

Additional File 6: Correlation between dystrophin transcript levels by real time qPCR and dystrophin protein amounts in TA muscle. We compared dystrophin protein levels by IF and WB with mRNA transcript levels at 30 days after one high dose PMO injection (800mg/kg). A) By RTqPCR, mdx-3 mouse shows the highest percent exon-skipping which translates to high dystrophin protein amounts as observed by B) WB and C) IF.
